# Muscle synergy structure and gait patterns in children with spastic cerebral palsy

**DOI:** 10.1111/dmcn.15068

**Published:** 2021-10-06

**Authors:** Marije Goudriaan, Eirini Papageorgiou, Benjamin R Shuman, Katherine M Steele, Nadia Dominici, Anja Van Campenhout, Els Ortibus, Guy Molenaers, Kaat Desloovere

**Affiliations:** ^1^ Department of Human Movement Sciences Vrije Universiteit Amsterdam Amsterdam the Netherlands; ^2^ Department of Rehabilitation Sciences KU Leuven Leuven Belgium; ^3^ Clinical Motion Analysis Laboratory University Hospitals Leuven Pellenberg Belgium; ^4^ Department of Mechanical Engineering University of Washington Seattle WA USA; ^5^ Henry M. Jackson Foundation for the Advancement of Military Medicine Bethesda MD USA; ^6^ Department of Development and Regeneration University of Leuven Leuven Belgium; ^7^ Department of Orthopedics University Hospitals Leuven Leuven Belgium

## Abstract

**Aim:**

To determine if muscle synergy structure (activations and weights) differs between gait patterns in children with spastic cerebral palsy (CP).

**Method:**

In this cross‐sectional study, we classified 188 children with unilateral (*n*=82) or bilateral (*n*=106) spastic CP (mean age: 9y 5mo, SD: 4y 3mo, range: 3y 9mo–17y 7mo; 75 females; Gross Motor Function Classification System [GMFCS] level I: 106, GMFCS level II: 55, GMFCS level III: 27) into a minor deviations (*n*=34), drop foot (*n*=16), genu recurvatum (*n*=26), apparent equinus (*n*=53), crouch (*n*=39), and jump gait pattern (*n*=20). Surface electromyography recordings from eight lower limb muscles of the most affected side were used to calculate synergies with weighted non‐negative matrix factorization. We compared synergy activations and weights between the patterns.

**Results:**

Synergy structure was similar between gait patterns, although weights differed in the more impaired children (crouch and jump gait) when compared to the other patterns. Variability in synergy structure between participants was high.

**Interpretation:**

The similarity in synergy structure between gait patterns suggests a generic motor control strategy to compensate for the brain lesion. However, the differences in weights and high variability between participants indicate that this generic motor control strategy might be individualized and dependent on impairment level.

## Abstract 2

This original article is commented by D'Avella et al. on pages 404–405 of this issue.

**Abbreviations**
sEMG: Surface electromyography
SnPM: Statistical non‐parametric mapping
tVAF_1_
: Total variance accounted for by one synergy

**What this paper adds**

- Synergy structure is similar between gait patterns in cerebral palsy (CP).
- Variability in synergy structure is high between children with CP.
- Greater impairment relates to more changes in synergy weights in CP.
- Synergies may reflect unique control strategies related to an individual’s impairments.
- Synergies and gait patterns can both reflect an individual’s motor control strategy.

Muscle synergies are defined as ‘consistent patterns of multi‐muscle coordination that generate a specific action’.^1^ They are thought to be regulated by central pattern generators in the spinal cord and the sensorimotor cortex.^1^, ^2^

Two basic muscle activity patterns (i.e. locomotor primitives) are already present in neonates during stepping movements.^3^, ^4^ With more walking experience, these locomotor primitives are modified and the number of muscle synergies increases.^3^, ^4^ These findings suggest that, although a part of the muscle synergies of gait appears to be present in the central nervous system (CNS) at birth, muscle synergies have the ability to adapt.^3^, ^4^

In individuals with a lesion in the CNS, such as children with cerebral palsy (CP), muscle synergies differ from their typically developing peers. These alterations are visible in a lower number of available synergies (i.e. decreased complexity of motor control). This decreased complexity of motor control has been quantified by a higher total variance accounted for by one synergy (tVAF_1_).^5^ In CP, tVAF_1_ is related to impairment level, with increased values of tVAF_1_ in children in higher Gross Motor Function Classification System (GMFCS) levels.^5^

Yu et al. reported significant differences in synergy activations between different GMFCS levels in CP, but not in synergy weights.^6^ Additionally, Steele et al. found differences in synergy activations and weights between different topographical types (e.g. unilateral vs bilateral).^5^

These previous studies grouped the children with CP based on either GMFCS level or affected body parts when studying differences in synergy structure (activations and weights).^5^, ^6^ While tVAF_1_ is considered representative of the functional level or topographical type, this might be less evident for synergy structure.^5^ For example, a child in GMFCS level II can walk in either a crouch or a jump gait pattern. For both patterns, tVAF_1_ could be similar, but the gait kinematics of these two patterns differ. The differences between gait patterns are related to the amount and timing of muscle activity during walking.^7^ Therefore, we expect that differences in synergy structure will be more evident when children with CP are grouped based on their gait patterns, instead of functional level or topographical type.

To test this hypothesis, we classified the gait patterns of children with CP between 3 and 17 years old (GMFCS levels I–III) according to six multiple joint gait patterns (i.e. combined motions of the different lower limb joints): drop foot, genu recurvatum, apparent equinus, crouch gait, jump gait, and true equinus. These most commonly observed pathological gait patterns in children with CP focus on deviations in the sagittal plane, and show a good intrarater reliability.^8^ An additional (seventh) ‘minor deviations pattern’ was added to accommodate for children with milder deviations.^8^, ^9^ These seven gait patterns have shown acceptance by the clinical CP community.^8^

## METHOD

This cross‐sectional retrospective study was approved by our local ethics committee (Commissie Medische Ethiek KU Leuven; S56036) under the Declaration of Helsinki.

### Participants

The database of the clinical motion analysis laboratory of Pellenberg was searched for previously classified 3D gait analyses of children with CP between 3 and 17 years old. Part of this database has already been published online.^9^ Inclusion criteria were: (1) children diagnosed with unilateral or bilateral spastic CP; (2) in GMFCS level I to III; (3) 3D gait analyses recorded after 2010 to reduce the influence of different data collection methods; (4) surface electromyography (sEMG) recordings of minimally one gait cycle with sufficient quality of at least one muscle representing the following groups: knee extensors, knee flexors, dorsiflexors, plantar flexors, and hip abductors; and (5) no history of orthopaedic or neurosurgery in the year before the 3D gait analyses or botulinum neurotoxin A injections 6 months before the 3D gait analyses. Only one 3D gait analysis per child was included and data from the most involved side, based on the outcomes of a standard clinical exam, were used for further analyses.

### Data collection

A 10 to 15 camera Vicon system (Vicon‐UK, Oxford, UK) and two force plates (AMTI, Watertown, MA, USA) collected marker trajectories and ground reaction forces. Markers were attached according to the lower body Plug‐in‐Gait model. We collected sEMG data from the rectus femoris, vastus lateralis, biceps femoris, medial hamstrings, tibialis anterior, medial gastrocnemius, soleus, and the gluteus medius, with a 16‐channel wireless sEMG system (Zerowire, Cometa, Italy) at 1000Hz or 1500Hz. The sEMG electrodes were attached according to the Seniam guidelines.^10^ Gait cycles and estimated pelvis and lower limb kinematics were defined using the Nexus software (Nexus 2.5. Vicon‐UK, Oxford, UK).

### Data analysis

#### Gait pattern classification

The number of gait cycles used for gait pattern classification varied between two and nine per participant. The sagittal plane motions of the pelvis, hip, knee, and ankle joints were compared to those of a typically developing sample of 57 children (mean age: 11y 4mo, SD: 4y 3mo, range: 4y 7mo–17y 1mo; 32 females). An experienced clinician classified kinematics of the children with CP by visual inspection of the separate, and averaged waveforms, of all gait cycles, plotted over the averaged waveforms (2 SD) of the typically developing sample. This experienced clinician followed gait patterns definitions from literature,^8^ and had an intrarater reliability of *k*=0.766 (unweighted; 95% confidence interval: 0.65–0.87). In case of doubt, a senior clinician was consulted.

The children with CP were classified into one of the six gait patterns: drop foot, genu recurvatum, apparent equinus, crouch gait, jump gait, or true equinus.^8^, ^11^ An additional pattern of ‘minor deviations’ was added, which represents sagittal plane kinematics that do not differ more than one standard deviation from the typically developing sample in at least 3 out of the 4 sagittal plane motions, but is still considered pathological.^9^

#### Synergy analysis

We selected four representative gait cycles to extract sEMG signals of the classified side. The sEMG signals were filtered with a sixth order Butterworth high‐pass filter with a cut‐off frequency of 20Hz. Quality of the high‐pass filtered sEMG signals was determined via visual inspection. The EMGs were classified with ‘good quality’ when there was obvious phasic activity and/or when low frequency or high amplitude artifacts were filtered out. Otherwise, EMG‐quality was considered ‘poor’. The sEMG signals were rectified and smoothed with a fourth order Butterworth lowpass filter with a 10Hz cut‐off frequency. We resampled the filtered sEMG signals of each gait cycle to 101 data points, representing 0% to 100% of a gait cycle, and concatenated the four gait cycles. The concatenated signal was normalized to its average amplitude.

We used weighted non‐negative matrix factorization in MATLAB (Mathworks Inc., Natick, MA, USA) to calculate muscle synergies with the following settings: 50 replicates, 1000 max iterations, and a 1×10^−6^ completion threshold.^12^, ^13^ Weighted non‐negative matrix factorization differs from regular non‐negative matrix factorization by assigning a weight to each data point.^12^, ^13^ Poor quality signals were assigned a weight of zero. Data of good quality were assigned a weight of one.

The inclusion of zero weighted signals has a limited effect on tVAF_1_ and synergy structure.^13^, ^14^ However, it allows for the inclusion of participants with poor quality or missing sEMG signals, thereby reducing the chance of selection bias.

By varying the number of synergies from one to five, we determined the minimum number of synergies required to explain 90% of variability in the data for each child based on the tVAF*_n_*. The tVAF*_n_* was calculated as:

$${\mathrm{tVAF}}_{n}=\left(1‐\frac{\left[\sum_{j}^{t}\sum_{i}^{m}{\left(\mathrm{error}\right)}^{2}\right]}{\left[\sum_{j}^{t}\sum_{i}^{m}{\left(\mathrm{sEMG}\right)}^{2}\right]}\right)$$

In this equation, *n* is the number of synergies (one to five), *m* is the number of muscles (eight), *t* is the number of data points of the concatenated signal (404), and error is the difference between the measured sEMG and reconstructed muscle activity signals computed using the corresponding synergy structure.^15^ Next, we used k‐means cluster analysis on the synergy weights to group similar synergies between the gait patterns.^16^ Additionally, we determined tVAF_1_ for each pattern.

We planned to include walking speed as a covariate in the statistical analyses because of its potential effect on muscle synergies.^17^ Hence, we extracted walking speed from the gait data and converted it to a non‐dimensional value.^18^ This method is used extensively in the evaluation of children with CP to compare speed across a wide spectrum of child size by normalizing by leg length.^19^

### Statistical analysis

We used the synergy weights from a pilot study to estimate the required sample size for a one‐way analysis of variance and post hoc *t*‐tests (GPower 3.1.9, Heinrich Heine University, Düsseldorf, Germany). The Dunn–Šidák correction was used to account for the multiple comparisons in our study population.

$$\alpha \_\mathrm{corr}=1‐{\left(1‐\;\alpha \right)}^{1/m}$$

In this equation, *α_*corr is the corrected alpha, *α* is 0.05, and *m* is the number of null hypotheses: (1) no differences in synergy activations, or (2) weights between gait patterns, and (3) no effect of walking speed on activations or (4) weights. This resulted an *α*=0.01.^20^ Power was set at 0.80. For the analysis of variance, the maximal effect size was 0.37 and the estimated sample size was 144. For the post hoc *t*‐test, maximal effect size was 1.42 with a sample size of 10 children. Descriptive statistics were used for differences in tVAF_1_ and walking speed.

We used statistical non‐parametric mapping (SnPM) to analyse the synergy activations (SPM1d version 0.4, available for download at http://www.spm1d.org/). The distribution of the synergy activations was determined with a built‐in function in statistical parametric mapping. Normality of the synergy weights and walking speed was checked with a Shapiro–Wilk test. None of the parameters had a normal distribution.

There is currently no non‐parametric analysis of covariance SnPM analysis available that could include walking speed as a covariate. Therefore, we first ran a non‐parametric one‐way analysis of variance (SnPM[F]). In case of a significant outcome, we used a post hoc non‐parametric two‐tailed, two‐sample *t*‐test (SnPM[t]) to assess potential differences in synergy activations between the gait patterns. Next, we used a non‐parametric canonical correlation analysis (SnPM[χ^2^]) to analyse the association between walking speed and synergy activations.

Potential differences in synergy weights were analysed with a non‐parametric analysis of covariance based on the Porter and McSweeney method.^21^ We used eta squared (*η* ^2^) as an effect size measure to determine how much of the variations in synergy weights between the gait patterns could be explained by differences in walking speed. In the below equation, SS_effect_ is the sum of squares for the effect that is studied and SS_total_ is the total sum of squares for all effects, errors, and interactions.

$$\eta^{2}=\frac{{\mathrm{SS}}_{\mathrm{effect}}}{{\mathrm{SS}}_{\mathrm{total}}}$$

In case of significant differences in weights between the gait patterns, we ran a post hoc Mann–Whitney *U* test.^21^

Since small clusters (e.g. of 1%) in an SnPM analysis can be unstable, we excluded clusters smaller than 5% of the gait cycle in length. For the SnPM analyses, the number of iterations was set at 10 000. All statistical analyses were performed in MATLAB.

## RESULTS

We selected 230 children with CP. Based on missing or poor quality sEMGs of the required muscle groups, we excluded 35 children. The 3D gait analyses of the remaining 195 children were grouped per gait pattern (Table 1). Detailed participant information can be found in Table S1 (online supporting information).

**Table 1:** Participant characteristics

	*n*	GMFCS level			Age, y:mo	Weight, kg	Height, m	Walking speed^a^	tVAF_1_
1. Minor deviations	34	I=33	II=1	III=0	10:11 (8:6–12:10)	33.3 (27.0–50.7)	1.46 (1.32–1.67)	0.42 (0.39–0.46)	0.72 (0.69–0.78)
2. Drop foot	16	I=13	II=3	III=0	10:4 (8:11–12:6)	22.2 (18.2–29.0)	1.21 (1.07–1.37)	0.44 (0.41–0.47)	0.73 (0.69–0.77)
3. Genu recurvatum	26	I=12	II=11	III=3	7:1 (5:7–10:0)	31.5 (23.5–46.2)	1.38 (1.24–1.60)	0.42 (0.38–0.43)	0.79 (0.74–0.81)
4. Apparent equinus	53	I=30	II=17	III=6	9:1 (6:4–12:2)	26.6 (20.4–37.0)	1.33 (1.17–1.50)	0.38 (0.32–0.46)	0.80 (0.75–0.85)
5. Crouch	39	I=12	II=16	III=11	11:2 (9:4–12:10)	34.7 (24.3–46.0)	1.42 (1.30–1.55)	0.33 (0.21–0.39)	0.80 (0.74–0.84)
6. Jump gait	20	I=6	II=7	III=7	5:9 (4:6–7:3)	17.9 (15.7–21.8)	1.10 (0.99–1.18)	0.42 (0.22–0.47)	0.83 (0.78–0.84)
7. True equinus^b^	7	I=2	II=3	III=2	5:8 (5:0–10:5)	18.4 (16.3–32.7)	1.19 (1.05–1.36)	0.43 (0.32–0.46)	0.81 (0.87–0.87)
Total	195	I=108	II=58	III=29	9:7 (6:4–11:11)	27.4 (20.2–38.6)	1.33 (1.16–1.51)	0.41 (0.32–0.46)	0.79 (0.73–0.83)

Values for age, weight, height, non‐dimensional walking speed, and tVAF_1_ are given in medians and the 25th–75th centiles.

^a^ Walking speed was converted to a non‐dimensional value according to Hof.^18^

^b^ The true equinus pattern was not included in the statistical analyses due the small sample size. GMFCS, Gross Motor Function Classification System; tVAF_1_, total variance accounted for by one synergy.

Of those 195 children, 170 children had good quality sEMGs of all eight muscles for all four gait cycles. The vastus lateralis was missing in 21 children (10% of all gait cycles), followed by the biceps femoris (*n*=17; 8% of all gait cycles), medial gastrocnemius (*n*=5; 2% of all gait cycles), rectus femoris (*n*=4; 1% of all gait cycles), and the medial hamstrings, tibialis anterior, soleus, and gluteus medius (all *n*=3; 1% of all gait cycles).

**Figure 1:**
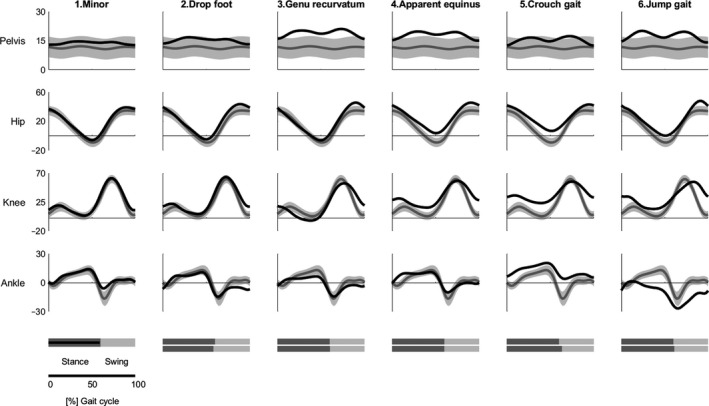
The different gait patterns identified in a previous systematic review,^8^ their sagittal plane joint angles, and average stance and swing duration (% gait cycle). The mean curve (1 SD) of reference sample of typically developing (TD) children is plotted in grey. On the bottom of the figure, stance and swing duration for the typically developing children are represented in light and dark grey respectively. The mean curve and mean stance phase duration of the children with cerebral palsy (CP) are plotted in black. The true equinus gait pattern was excluded because it did not meet the sample size requirements.

The most frequent gait pattern was apparent equinus (*n*=53), followed by crouch gait (*n*=39), minor deviations (*n*=34), genu recurvatum (*n*=26), jump gait (*n*=20), drop foot (*n*=16), and true equinus (*n*=7). The true equinus group did not meet the sample size requirements and was excluded, leaving 188 children for subsequent analyses (mean age: 9y 5mo, SD: 4y 3mo, range: 3y 9mo–17y 7mo; 75 females; GMFCS level I: 106, GMFCS level II: 55; GMFCS level III: 27). An overview of the kinematics of the gait patterns is given in Figure 1, as well as representative videos of each gait pattern (see Videos [Link], [Link], [Link], [Link], [Link], [Link], [Link], online supporting information).

The reported parameters are represented in medians and interquartile ranges. The children who were less impaired (minor deviations and drop foot) had the lowest tVAF_1_ (0.72 [0.08] and 0.73 [0.07] respectively) and the fastest walking speed (0.42 [0.05] and 0.44 [0.06] respectively).

**Figure 2:**
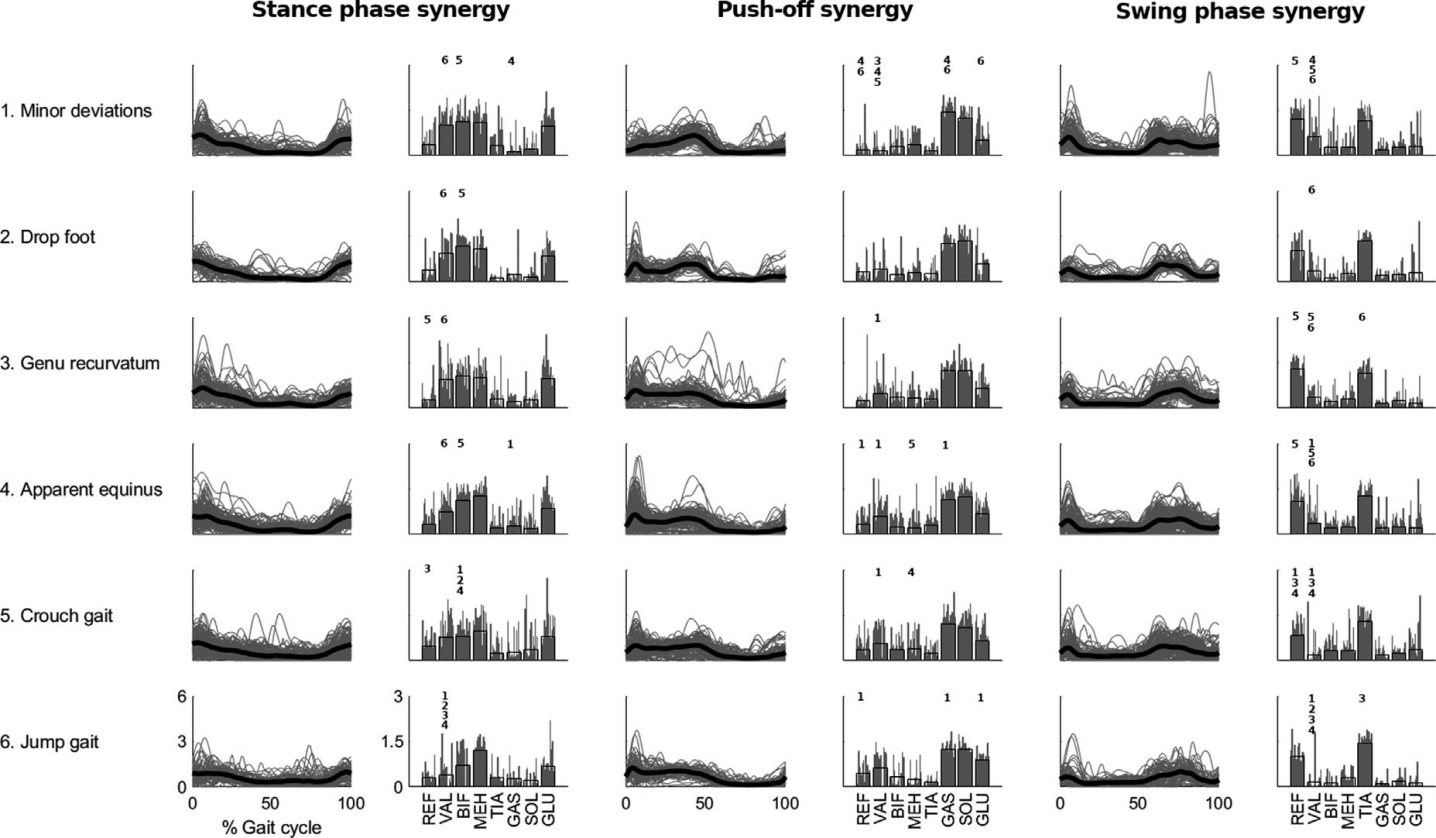
Synergy activations and weights per gait pattern. Synergy weights for each child and synergy activations for each of the four gait cycles per child are represented in grey and as the mean activation and weight for each pattern (black) as a function of the normalized gait cycle. Significant differences between the individual patterns (post hoc analyses) are indicated with the numbers of the significantly different patterns. REF, rectus femoris; VAL, vastus lateralis; BIF, biceps femoris; MEH, medial hamstrings; TIA, tibialis anterior; GAS, gastrocnemius; SOL, soleus; GLU, gluteus medius.

For all six gait patterns, 90% of the variability in the sEMG patterns could be explained by three synergies. From the k‐means cluster analysis, the following three synergies were identified: a stance phase, push‐off, and swing phase synergy (Fig. 2). Individual outcomes of the cluster analysis can be found in Table S1.

No significant differences were found between the gait patterns regarding synergy activations (stance phase synergy: SnPM[F]=4.782, push‐off synergy: SnPM[F]=4.729, swing phase synergy: SnPM[F]=4.792). We found between‐pattern differences in synergy weights of all eight muscles for all synergies (analysis of covariance). Walking speed was associated with differences in the synergy weights of the rectus femoris (all three synergies), medial gastrocnemius (push‐off and swing phase synergies), and vastus lateralis (swing phase synergy). Yet, the effect sizes for both walking pattern and speed accounted for less than 15% of the interindividual variance in synergy weights (Table 2). Significant differences in synergy weights are only described for the main contributors of each synergy (see Fig. 2 and Table S2, online supporting information, for further details).

**Table 2:** Effect sizes (*η* ^2^) indicating how much of the differences in synergy weights can be explained by gait pattern or walking speed

	Rectus femoris	Vastus lateralis	Biceps femoris	Medial hamstrings	Tibialis anterior	Gastrocnemius	Soleus	Gluteus medius
Stance phase synergy								
Pattern	0.119^c^	0.123^c^	0.117^c^	0.140^c^	0.136^c^	0.133^c^	0.135^c^	0.131^c^
Speed	0.038^b^	0.017	0.011	<0.001	0.010	0.012	0.003	0.005
Push‐off synergy								
Pattern	0.120^c^	0.126^c^	0.141^c^	0.140^c^	0.142^c^	0.118^c^	0.127^c^	0.132^c^
Speed	0.060^c^	0.005	0.001	0.016	0.005	0.048^b^	0.019	0.007
Swing phase synergy								
Pattern	0.091^b^	0.089^b^	0.136^c^	0.142^c^	0.133^c^	0.144^c^	0.142^c^	0.143^c^
Speed	0.107^c^	0.037a	<0.001	0.004	0.031	0.040^b^	0.011	0.003

Significant effects are indicated as follows: ^a^ *p*<0.01; ^b^ *p*≤0.005; ^c^ *p*≤0.001.

The main contributors to the stance phase synergy were the vastus lateralis, biceps femoris, medial hamstrings, and the gluteus medius. The post hoc analyses showed that the vastus lateralis weights were significantly lower in the jump gait pattern (0.22 [0.64]) when compared to all other patterns (minor deviations: 1.06 [0.72], *p*<0.001; drop foot: 1.06 [0.89], *p*<0.01; genu recurvatum: 0.89 [1.01], *p*<0.005; apparent equinus: 0.74 [0.72], *p*<0.005), except crouch gait (0.64 [0.92]). The biceps femoris weights of the crouch gait pattern (0.99 [0.63]) were significantly lower than in the minor deviations (1.14 [0.76], *p*<0.01), drop foot (1.31 [0.41], *p*<0.005), and apparent equinus (1.22 [0.47], *p*<0.001) patterns (Fig. 2).

The push‐off synergy was characterized by activity of the plantar flexors. The medial gastrocnemius weights of the apparent equinus (1.21 [0.37]) and jump gait (1.25 [0.31]) patterns were significantly lower than for the minor deviations pattern (1.48 [0.36]; *p*<0.001 and *p*<0.01 respectively, Fig. 2).

The rectus femoris and tibialis anterior were mostly active in the swing phase synergy. Rectus femoris weights were lower in the crouch gait pattern (0.82 [0.68]) when compared to the minor deviations (1.40 [0.69], *p*<0.001), genu recurvatum (1.37 [0.45], *p*<0.001), and apparent equinus patterns (1.11 [0.53], *p*<0.01). Tibialis anterior weights were higher in the jump gait pattern (1.51 [0.32]) than in the genu recurvatum pattern (1.20 [0.43]; *p*<0.005) (Fig. 2).

## DISCUSSION

We expected to find differences in synergy activations and weights between gait patterns in children with CP, while considering variations in walking speed. However, there was a high similarity in synergy structure between the gait patterns. Although there were differences in synergy weights, these were not clearly related to the gait pattern or walking speed. Additionally, we noticed a high variability in both synergy activations and weights between our participants.

In stroke survivors, the red nucleus in the midbrain is thought to (imperfectly) compensate for lesions in the pyramidal tracts.^22^, ^23^ The red nucleus and the rubrospinal tract are involved in simple, stereotyped flexion‐extension movements.^22^, ^23^ The imperfect compensation of the brain lesion by the red nucleus into more generic movement patterns could explain the similarity in synergy structure between gait patterns.

Muscle synergies are repeatable between days and across walking speeds in CP.^17^, ^24^ Therefore, we believe that the differences in synergy weights between the gait patterns and the high variability in synergy structure between our participants are associated with individualized motor control strategies. An individual’s strategy depends on brain lesion location and severity,^25^, ^26^ combined with the neural capacity of the CNS to compensate for the lesion.^22^, ^23^ How these different motor control strategies relate to gait kinematics needs further research.

The ability to selectively activate the muscles required for a certain task (e.g. selective motor control) can be considered a reflection of the neural capacity of the CNS. Clinically, selective motor control has been quantified under (semi‐)static conditions with the selective control assessment of the lower extremity and with coactivation measures.^27^, ^28^ During walking, selective motor control can be assessed with tVAF_1_.^17^, ^24^ Synergy structure reflects the level of coactivation of all measured muscles and could provide unique insights into an individual’s chosen motor control strategy.

In CP, higher impairment levels are associated with a decrease in selective motor control with progressively more proximal joint involvement.^29^ Indeed, the children who were more severely impaired (crouch and jump gait) had higher tVAF_1_ values and altered kinematics in both distal and proximal joints (Table 1, Fig. 1). For synergy structure, the association between selective motor control and the more impaired gait patterns was less obvious, although more differences in synergy weights were seen with increasing impairment levels.

A limitation of our study is that the SnPM analyses in MATLAB do not allow assessment of heteroscedasticity. Additionally, the weights of the tibialis anterior showed heteroscedasticity in the stance phase synergy, and the rectus femoris and vastus lateralis weights in the push‐off synergy.^30^ Since we did not find significant differences between gait patterns in synergy activations nor the tibialis anterior weights of the first synergy, we expect that a violation of homoscedasticity would have a limited effect on the outcomes. However, significant differences in rectus femoris and vastus lateralis weights between gait patterns in the push‐off synergy should be interpreted with some caution.

This and prior research suggests the CNS compensates for brain lesion by using simplified movement patterns. However, within these generic movement patterns, our children with CP showed individualized motor control strategies. Brain lesion location and severity, in combination with the neural capacity of the CNS to compensate for this brain lesion, are expected to dictate this strategy. How these motor control strategies interact with musculoskeletal impairments and relate to gait kinematics still needs to be determined. However, it seems that tVAF_1_ (neural capacity), synergy structure (employed motor control strategy), and gait kinematics (the results of this strategy) show, on different levels, how successfully the CNS compensates for an individual’s brain lesion.

## CONFLICT OF INTEREST

The authors declare that the research was conducted without any commercial or financial relationships.

## Supporting information


**Table S1:** Contains all the data that was used in this study, including brain lesion type, and clinical outcome measures

**Table S2:** Contains all statistical outcomes from the analyses used in the study

**Video S1:** Minor deviations pattern.

**Video S2:** Drop foot pattern.

**Video S3:** Genu recurvatum pattern.

**Video S4:** Apparent equinus pattern.

**Video S5:** Crouch gait pattern.

**Video S6:** Jump gait pattern.

**Video S7:** True equinus pattern.

## Data Availability

The data that support the findings of this study are openly available in [figshare] at http://doi.org/10.6084/m9.figshare.13123175.
